# Influence of the Adaptive Torque Control Motion on the Ability of Neolix EDMax to Reach Working Length When Used as a Single Shaping File—An In Vitro Study

**DOI:** 10.3390/dj13060262

**Published:** 2025-06-12

**Authors:** Vlad Mircea Lup, Carlo Gaeta, Ashkan Tavakkoli, Andreas Louloudiadis, Simone Grandini, Gabriela Ciavoi

**Affiliations:** 1 Doctoral School of Biomedical Sciences, University of Oradea, 410 087 Oradea, Romania; 2 Unit of Endodontics and Restorative Dentistry, Department of Medical Biotechnologies, University of Siena, 53100 Siena, Italy; ashkan.tavakkoli@student.unisi.it (A.T.); grandini@unisi.it (S.G.); 3 Private Practice, London GU1 2AA, UK; alouloud@gmail.com; 4 Faculty of Medicine and Pharmacy, University of Oradea, 410 087 Oradea, Romania; gciavoi@uoradea.ro

**Keywords:** dental equipment, root canal preparation, rotation, tooth apex, single file, working length

## Abstract

**Objectives:** The aim of this study is to investigate how Adaptive Torque Control motion influences the shaping efficiency of Neolix EDMax (Neolix SAS, Évron, France) and its ability to reach working length with or without a pre-existing glide path. **Methods:** A total of 90 endo training blocks with an S-shape curvature were divided into three groups based on the kinematics and preparation phase: the control group, where the Neolix EDMax (Neolix SAS, Évron, France) was used for shaping after a glide path was established; the no glide path group, where the Neolix EDMax (Neolix SAS, Évron, France) was used for shaping without a glide path; and the Adaptive Torque Control group, where the Neolix EDMax (Neolix SAS, Évron, France) was used for shaping without a glide path but in an Adaptive Torque Control motion. The time for shaping, the instrument passes, and the ability to reach working length were recorded and analyzed using a one-way Anova and Tukey’s HSD post hoc test. **Results:** Establishing a glide path helped the shaping file to reach working length faster and in fewer passes when compared with the no glide path group, but the Adaptive Torque Control group was able to perform even better than the control group despite not having a pre-established glide path. **Conclusions:** The Adaptive Torque Control motion on continuous rotation instruments does impact their performance. Combining the efficiency of continuous rotation and the safety of reciprocation, this type of motion had a significant effect on the ability to shape the simulated root canal even in the presence of a double curvature and without a pre-established glide path.

## 1. Introduction

Since the introduction of nickel-titanium (Ni-Ti) instruments, the shaping of root canals has improved in terms of efficiency and predictability [1]. Ni-Ti instruments have been shown to have superior elastic flexibility while twisting and bending, as well as enhanced mechanical properties compared to stainless steel files [2]. Improvements in metallurgy allowed the development of a wide range of instruments with unique designs, resulting in a better control of iatrogenic errors and an improvement in root canal shaping, making it easier, faster, and with better clinical outcomes [3].

Despite the continuous improvement in Ni-Ti metallurgy and manufacturing technologies, the lifespan of these instruments is related to the stress levels they can endure, so instrument failure might occur [4]. These failure mechanisms have been intensively studied, and one of the causes has been shown to be torsional failure, which happens when the tip of the instrument binds inside the root canal while the rest of the instrument keeps rotating, generating a level of stress greater than the strength of the material [5].

In order to try to reduce torsional failures, torque control functions have been added to endodontic motors. It is known that, when a file starts rotating inside a root canal, it generates torque [4]. In endodontics, torque refers to the force needed to rotate the instrument. When using a Ni-Ti instrument, the motor must ensure a constant rotational speed (without accelerations or decelerations) in all conditions, regardless of root canal curvature or calcification, in order to maintain a decent stress condition for the instrument [6].

Another factor that can influence the generated torque is the kinematics of the Ni-Ti files [4]. Every time a file that is constricted inside a root canal cuts dentin, a certain degree of torsional deformation develops around its axis. If this deformation can be maintained under the limits of plastic deformation, structural changes should not appear [7]. The idea of limiting the angle of rotation under the elastic limit has led to a movement best described as partial or asymmetrical reciprocation [7]. As a result, reciprocating files came to the market with a motion that has greater cutting counterclockwise (CCW) angles and smaller disengaging clockwise (CW) angles. However, there are authors suggesting that a forward reciprocating motion with greater CW angles than CCW angles might expand the lifespan of files designed for continuous rotation [8,9].

A major innovation in endodontic motors was the implementation of Adaptive Torque Control (ATC) mode, in which clinicians can manage the stress on the rotary instruments. Essentially, when a pre-set torque value (usually under 1 N/cm) is reached, the instrument goes into a forward reciprocation following the pre-set angles in order to reduce accumulated stress, instead of going into auto reverse. Once the measured torque goes below the pre-set levels, the instrument will return to a continuous rotation.

Beside kinematics innovations, different instrument production methods have been developed, one of which was electric discharge machining (EDM) [10]. This manufacturing process uses local vaporization of the metal, thus preventing the formation of microcracks and theoretically increasing the cutting efficiency, flexibility, and cyclic fatigue resistance of instruments [10,11].

The first instrument on the market produced with EDM was an orifice opener named Initial (Neolix SAS, Évron, France) in 2012. In 2013, the same company produced a series of rotary instruments, the Neoniti system (Neolix SAS, Évron, France). After ten years, in 2023, a new rotary system was introduced, the EDMax system (Neolix SAS, Évron, France). Compared to Neoniti, the EDMax presents sharp cutting edges and a hardened and abrasive surface in order to improve their efficiency and shaping ability.

Currently, there are studies showing the abilities of Reciproc (WDV, Munchen, Germany) [12,13,14,15,16] and WaveOne (Dentsply Sirona, Ballaigues, Switzerland) [17,18] to reach working length even without a preestablished glide path, but there is a lack of studies researching instruments designed for continuous rotation.

Therefore, the aim of this study is to investigate how ATC motion influences the shaping efficiency of Neolix EDMax and if it has an impact on the ability to reach working length with or without a pre-existing glide path. We hypothesize that, with the aid of ATC, instruments used for continuous rotation will perform better and with fewer structural changes compared to pure rotation.

## 2. Materials and Methods

For this in vitro study, we utilized resin training blocks for the main reason that they can be easily standardized regarding length, taper, curvature, and hardness, parameters than cannot be standardized in extracted teeth.

A power calculation was performed using G Power 3.1 (Heinrich Heine University, Düsseldorf, Germany). For a one-way analysis of variance (ANOVA), the software indicated that a total sample size of 81 samples (27 per group) would be required to achieve a statistical power of 0.8 with an alpha of 0.05 and an effect size (f = 0.35).

Therefore, for this study, a total of 90 (*n* = 90) endodontic training blocks (Dentsply Sirona, Ballaigues, Switzerland) presenting an S-shaped root canal were used. The length of the simulated canals was 16 mm, and the taper was 2%. The first curve had a 30° angle, a radius of 3 mm, and was situated at 8 mm from the terminus, whereas the second curvature had a 25° angle, a radius of 2 mm, and was located at 2 mm from the terminus.

The blocks were randomly divided between 3 groups (*n* = 30 each) and prepared by the same single operator with fifteen years of experience, as follows:

Control Group (CTR): All 30 blocks in this group were scouted with a new #10 K-file (Dentsply Sirona, Ballaigues, Switzerland) by the same operator. After that, a glide path was established according to the manufacturer protocol using new EDMax Glide path files (Neolix SAS, Évron, France) at 500 rotations per minute (rpm) and a torque value of 1.5 N/cm, as recommended. The shaping was done by the same operator using new 25/06 EDMax Shaping files (Neolix SAS, Évron, France) at 500 rpm and a torque value of 1.5 N/cm in several passes until working length was reached or the file separated. The endodontic motor used was Endo Radar Pro (Woodpecker Medical Instrument Co., Ltd., Guilin, China). The irrigation protocol of Irrigation, Recapitulation, Irrigation (IRI) was performed with saline solution between every three instrument passes to remove debris accumulation.

No Glide Path Group (NGP): The 30 blocks in this group were scouted with a new #10 K-file (Dentsply Sirona, Ballaigues, Switzerland) by the same operator. After that, the shaping was performed directly by the same operator with new 25/06 EDMax Shaping files (Neolix SAS, Évron, France) at 500 rpm and a torque value of 1.5 N/cm in several passes until working length was reached or the file separated. The endodontic motor used was Endo Radar Pro (Woodpecker Medical Instrument Co., Ltd., Guilin, China). The IRI protocol was performed with saline solution between each three instrument passes to remove debris accumulation.

Adaptive Torque Control Group (ATC): The 30 blocks in this group were scouted with a new #10 K-file (Dentsply Sirona, Ballaigues, Switzerland) by the same operator. After that, the shaping was performed directly by the same operator with new 25/06 EDMax Shaping files (Neolix SAS, Évron, France) in ATC mode. The motor used was Endo Radar Pro (Woodpecker Medical Instrument Co., Ltd., Guilin, China), and the program was the Adaptive Torque Reverse (ATC mode in the Endo Radar Pro system library) at a speed of 500 rpm with a preset torque value of 0.8 N/cm, a preset forward cutting angle of 120 degrees, and a preset reverse disengaging angle of 30 degrees. The instrument would use a continuous rotation motion until the measured torque value exceeded 0.8 N/cm (the preset value), then would go into a forward reciprocation motion using the preset angles until the measured torque was lower than 0.8 N/cm, the point in which the movement would change to continuous rotation again. The irrigation between each three instrument passes was done with saline solution and the IRI protocol to remove debris accumulation.

**Figure 1:**
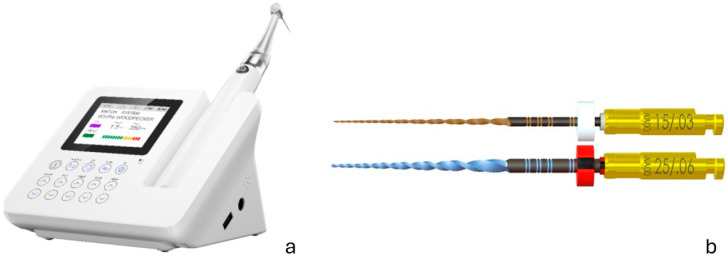
Materials used for this study: (**a**). Endo Radar Pro Endodontic Motor; (**b**). Neolix EDMax Glidepath and Neolix EDMax Shaper 25/06.

All rotary instruments needed for this study (30 EDMax Glidepath files and 90 25/06 EDMax files) were provided by Neolix with the following batch numbers: 20230907 (EDMax Glidepath, Neolix SAS, Évron, France) and 20220122 (EDMax Shaper 25/06, Neolix SAS, Évron, France). The endodontic motor and the files used for this study are depicted in Figure 1.

The shaping preparation time in seconds for every block in each group was recorded with a Seiko high precision chronometer (Seiko Holding Corporation, Japan). The time for cleaning the flutes and irrigation protocol was not recorded, meaning the chronometer was started every time the instrument entered the simulated canal and paused the moment the instrument left the canal. In order to avoid discrepancies in measuring the shaping time, an assistant blinded to the groups was trained to start and pause the chronometer as needed.

The ability of the EDMax 25/06 instrument to reach working length, the misshapes such as ledge formation, and the incidence of instrument separation were also recorded and analyzed.

All statistical analyses were performed using Stata/MP 18 (Statacorp LP Lakeway drive, TX, USA). Descriptive statistics, including mean and standard deviation, were calculated for shaping time, number of pecking motions (NPM), and average time per peck across the three experimental groups (CTR, NGP, and ATC). The normality of residuals was verified using the Shapiro–Wilk test applied to the residuals of the ANOVA model. Since the assumption of normality was met, a one-way ANOVA was used to compare shaping time and NPM among the groups. When significant differences were found, Tukey’s HSD post hoc test was applied to determine which specific groups differed significantly. Working length achievement (RFWL) was reported using descriptive statistics only, as the study was not powered to detect inferential differences in binary outcomes.

## 3. Results

A total of 87 simulated canals were included in the analysis after excluding three failures in the NGP group. The descriptive statistics for shaping time, number of pecking motions (NPM), average time per peck, and working length (WL) achievement are presented in Table 1. The ATC group demonstrated the shortest mean shaping time (14.75 ± 2.18 s), followed by the CTR group (16.66 ± 2.66 s) and NGP group (20.48 ± 3.15 s). The number of pecking motions followed a similar trend, with ATC requiring the fewest (9.27 ± 1.31) and NGP the most (15.93 ± 2.74). All groups achieved 100% WL success except for the NGP group, which had three cases of failure (90% success).

A one-way ANOVA test revealed a statistically significant effect of instrumentation technique on shaping time (F (2,84) = 33.42, *p* < 0.001), indicating that the type of motion significantly influenced efficiency. The results of the Tukey Honestly Significant Difference (HSD) post hoc test for shaping time identified which specific group pairs differed significantly. All comparisons show significant differences, with the largest contrast between ATC and NGP (Table 2).

**Figure 2:**
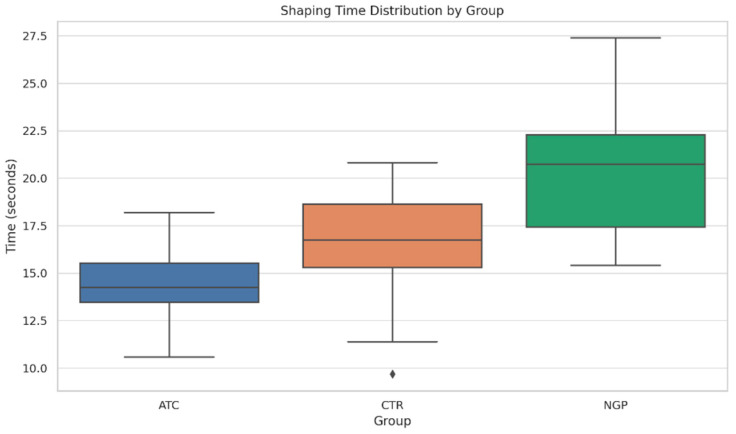
Boxplot of shaping time by group. The diamond mark represents the individual sample whose shaping time falls more than 1.5 × IQR beyond the lower quartile.

The boxplot of shaping time (Figure 2) displays the distribution of shaping time (in seconds) across groups. The ATC group showed the lowest median shaping time and a relatively tight distribution, suggesting consistent performance.

The ANOVA for NPM also showed a highly significant group effect (F (2,84) = 77.93, *p* < 0.001) (Table 3). A highly significant F value supports the hypothesis that instrumentation method affects the number of motions used during shaping. Tukey’s HSD post hoc comparison for NPM showed that the ATC group required significantly fewer pecking motions than the NGP group (*p* < 0.001) and numerically fewer motions than the CTR group, although this difference did not reach statistical significance (*p* = 0.105). These findings support the shaping efficiency of torque-adaptive motion, with ATC showing a consistent trend toward reduced mechanical effort compared to both other groups.

**Figure 3:**
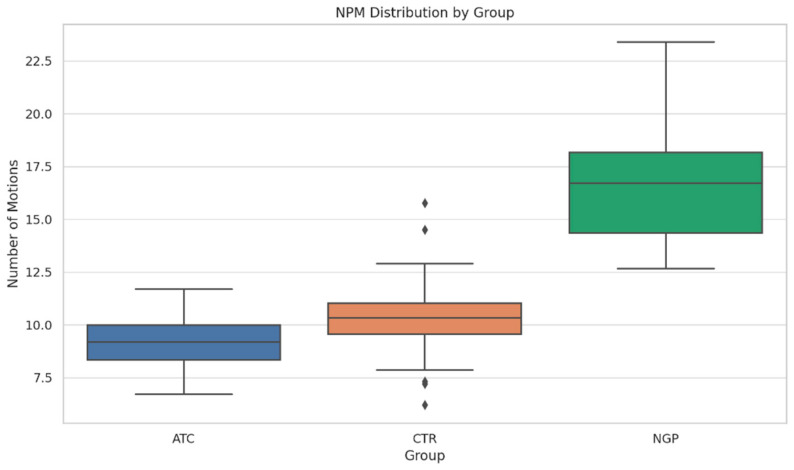
Boxplot of the number of pecking motions by group. The diamond mark represents the individual sample whose shaping time falls more than 1.5 × IQR beyond the lower quartile.

The boxplot of pecking motions by group (Figure 3) shows that the ATC group had the fewest and most consistent pecking motions. The NGP group exhibited a higher and more variable count, reinforcing the advantage of ATC in improving procedural efficiency.

**Figure 4:**
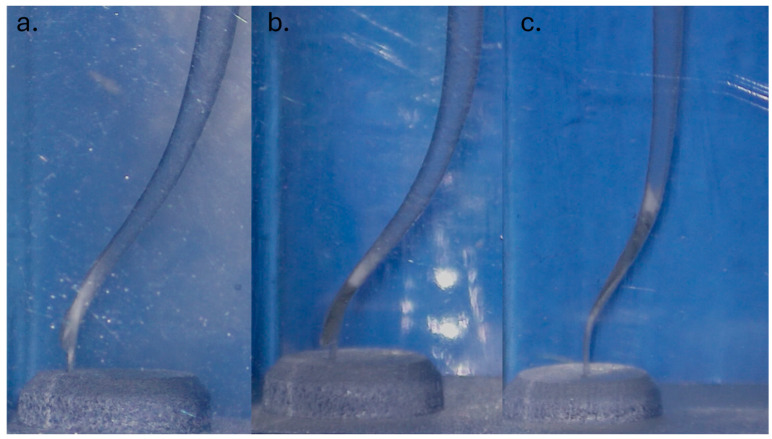
Samples who failed to reach the working length: (**a**). NGP 8 ledge formation; (**b**). NGP 17 instrument separation; (**c**). NGP 28 instrument separation.

Regarding the ability of the Neolix EDMax Shaper to reach working length, the CTR and ATC groups presented a 100% success rate. In the NGP group, the EDMax shaper failed to reach working length in three samples, recording one ledge formation (Figure 4a) and two instrument separations (Figure 4b,c).

## 4. Discussion

Although endodontic training blocks have their limitations when used in in vitro studies due to the difference between resin and dentin, they are ideal for standardizing experimental conditions [19]. However, two main factors need to be taken into consideration. First, the physical features of resin blocks do not match those of dentin, especially regarding microhardness; second, resin blocks have different thermal properties compared to natural dentin. Due to the fact that the hardness of resin is about half of that observed in natural dentin, all studies that used the shaping of resin blocks were more likely to present some form of deviation or canal transportation. That is why the results should be interpreted with caution, as they can slightly differ if extrapolated to natural teeth. In this study, we used these blocks in order to determine if the ATC motion for continuous rotation instruments has any influence on the shaping parameters and the ability of said instruments to reach working length with or without a pre-established glide path.

ATC motion consists of alternating continuous rotation with a forward reciprocation motion depending on the torque value read by the endodontic motor, combining the efficiency of continuous rotation with the safety of reciprocation [20]. An asymmetrical reciprocation movement was first described by Yared, who used a ProTaper Universal F2 in a forward reciprocation [7], and since then, authors have acknowledged the benefits of forward reciprocation in expanding the lifespan of Ni-Ti instruments designed for use in continuous rotation [9].

Even though Neolix EDMax is not designed to be used as a single file, it is meant to be a successor of the Neoniti (Neolix, SAS), which outperformed other instruments [21,22] with improved efficiency and shaping ability. In order to properly assess the impact of ATC on continuous rotation instruments, the EDMax 25/06 was used as a single file with a pre-established glide path according to the manufacturer protocols, without a glide path following manufacturer settings, and in ATC motion with reciprocation angles set based on the instrument’s cross-section design.

When comparing shaping times, the NGP group exhibited the longest time (20.48 ± 3.15 s), as expected. Compared to the CTR group, the Neolix EDMax 25/06 had to shape an unenlarged simulated canal, resulting in shorter strokes and extended time. Similar conclusions were made in other studies regardless of the type of motion, continuous rotation [23] or reciprocation [24], and in reviews [25]. However, the ATC group was able to reach working length faster compared to the CTR group (14.75 ± 2.18 s compared to 16.66 ± 2.66 s). An explanation for these results could be that, even if the CTR group had a pre-established glide path, after the first curvature, the shaping instrument advanced slower in continuous rotation with a low torque compared with the forward reciprocation of the ATC group, which was able to perform faster strokes and thus performed faster even with the “loss of time” for instrument disengagement. There are studies that research shaping time with adaptive motion versus continuous rotation, but not ATC motion in particular. One study [23] found that adaptive motion reaches working length faster on a smaller glide path but not on a larger one, while another study [26] concludes the opposite, but places adaptive motion as being faster in general on all glide path sizes.

Regarding the number of pecking motions, the NGP group needed the most passes in order to reach working length (15.93 ± 2.74 vs 10.4 ± 2.18 on CTR group). It is clear that a large instrument advances more slowly in a narrow canal than in one pre-enlarged by a glide path, data also supported by previous literature [18]. Although the ATC group required fewer pecking motions than the CTR group (9.27 vs. 10.4), this difference did not reach statistical significance (*p* = 0.105). This may be explained by the fact that, in the double curvature portion, the forward reciprocation of ATC allows the instrument to advance more effectively than continuous rotation, potentially reducing the need for additional pecking motions, further supporting the motion’s shaping efficiency.

In terms of the ability to reach working length, the CTR group returned no surprising results. Even though Neolix EDMax was not meant to be used as a single file, when a glide path was present according to the manufacturer’s recommendations, the instrument had no problems negotiating a double curvature and reached working length in all samples without incidents. The benefits of creating a glide path are well known and described in current literature reviews [27,28].

In the NGP group, where no glide path was present, the Neolix EDMax failed to reach the working length in three samples, recording one ledge formation and two instrument separations.

**Figure 5:**
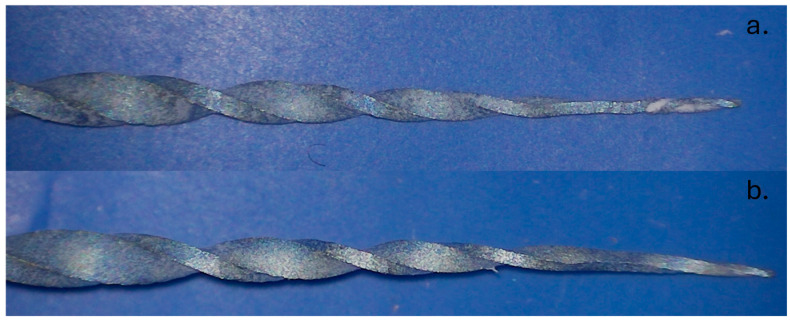
File deformations recorded before fracture: (**a**) NGP 17 file deformation; (**b**) NGP 28 file deformation.

The ledge and obvious transportation could account for the softness of the resin block compared to natural dentin. Due to the fact that no glide path was present, the tip of the instrument was severely constricted in the second curve, causing the rest of the instrument to cut more on the outside of the curve. Instrument separations can be easily avoided in an in vivo scenario because, before the separations occurred, both instruments presented deformations (Figure 5) that were spotted during the irrigation protocol when they were pulled out, cleaned, and inspected. However, since the purpose of this study was to analyze if Neolix EDMax can reach working length regardless of its condition, the instruments could not be discarded and had to be allowed to work until they reached working length or they separated.

In the ATC group, all instruments managed to reach working length without incidents like ledge formation or file separation. Although there were instruments that exhibited minor deformation, none of them separated due to the safety of the forward reciprocation motion, which kept the deformation below the elastic limit of the instruments [7]. The authors are unaware of similar studies regarding ATC motion on rotary files used without a glide path in order to compare the results in this section.

Overall, the findings of this study support the use of ATC as a shaping protocol that combines the speed of continuous rotation with the safety advantages of reciprocation. Even when used without a glide path, ATC showed excellent shaping efficiency and reliability, comparable to or better than the standard multi-step protocol. These results may have important implications for clinical protocols, suggesting that ATC-equipped motors can safely and efficiently shape canals even in the absence of a glide path, potentially simplifying procedures in selected cases. Future research of this topic is needed to see if the same results apply on natural dentin, and if different preset angles and torque values have an impact on the performance of the instruments depending on their cross-section.

## 5. Conclusions

Within the limitations of this in vitro study, it can be concluded that ATC motion on continuous rotation instruments does indeed impact their performance. Combining the efficiency of continuous rotation and the safety of reciprocation, this type of motion had a significant effect on the ability to shape the simulated root canal even in the presence of a double curvature and without a pre-established glide path. More studies are required to further investigate the impact of preset angles of reciprocation on the cross-section design of the instruments.

## Figures and Tables

**Table 1:** Descriptive statistics for shape time, NPM, and RFWL in CTR, NGP, and ATC groups.

		Shape Time	NPM	Avg. Time/Peck	RFWL		
Group	*n*	Mean ± SD (s)	Mean ± SD (s)	Mean ± SD (s)	Success	Failure	Success%
ATC	30	14.75 ± 2.18	9.27 ± 1.31	1.59 ± 0.11	30	0	100
CTR	30	16.66 ± 2.66	10.4 ± 2.18	1.63 ± 0.21	30	0	100
NGP	30	20.48 ± 3.15	15.93 ± 2.74	1.29 ± 0.1	27	3	90

**Table 2:** Shaping time by instrumentation group.

Group	Mean ± SD (s)	ANOVA
ATC	14.75 ± 2.18 ^a^	F (2,84) = 33.42, *p* < 0.001
CTR	16.66 ± 2.66 ^b^	
NGP	20.48 ± 3.15 ^c^	

Note: Means sharing a letter are not significantly different (Tukey’s HSD, *p* < 0.05).

**Table 3:** Number of pecking motions (NPM) by instrumentation group.

Group	Mean ± SD (s)	ANOVA
ATC	9.27 ± 1.31 ^a^	F (2,84) = 77.93, *p* < 0.001
CTR	10.4 ± 2.18 ^ab^	
NGP	15.93 ± 2.74 ^c^	

Note: Groups sharing a letter are not significantly different; groups with different letters differ at *p* < 0.05 (Tukey HSD).

## Data Availability

Dataset available on request from the authors.
